# Inhibition of Glioma Development by ASCL1-Mediated Direct Neuronal Reprogramming

**DOI:** 10.3390/cells8060571

**Published:** 2019-06-11

**Authors:** Xueyan Cheng, Zijian Tan, Xiao Huang, Yimin Yuan, Shangyao Qin, Yakun Gu, Dan Wang, Cheng He, Zhida Su

**Affiliations:** 1 Center for Brain Disorders Research, Capital Medical University; Center of Neural Injury and Repair, Beijing Institute for Brain Disorders, Beijing 100020, China; cxy19930528@163.com (X.C.); yakungu2015@163.com (Y.G.); 2 Institute of Neuroscience, Key Laboratory of Molecular Neurobiology of Ministry of Education and the Collaborative Innovation Center for Brain Science, Second Military Medical University, Shanghai 200433, China; zjiantan@163.com (Z.T.); huangxiao361@163.com (X.H.); yym3535@163.com (Y.Y.); shangyao_qin@163.com (S.Q.); Danwang2011@126.com (D.W.)

**Keywords:** ASCL1, reprogramming, glioma cells, neurons

## Abstract

Direct conversion of non-neural cells into induced neurons holds great promise for brain repair. As the most common malignant tumor in the central nervous system, glioma is currently incurable due to its exponential growth and invasive behavior. Given that neurons are irreversible postmitotic cells, reprogramming glioma cells into terminally differentiated neuron-like cells represents a potential approach to inhibit brain tumor development. We here show that human glioma cells can be directly, rapidly and efficiently reprogrammed into terminally differentiated neuron-like cells by the single transcription factor ASCL1 (Achaete-scute complex-like 1, also known as MASH1). These induced cells exhibit typical neuron-like morphology and express multiple neuron-specific markers. Importantly, ASCL1-mediated neuronal reprogramming drives human glioma cells to exit the cell cycle and results in dramatic inhibition of proliferation, both in vitro and in vivo. Taken together, this proof-of-principle study demonstrates a potential strategy for impeding brain tumor development by ASCL1-induced direct neuronal reprogramming.

## 1. Introduction

In the central nervous system, gliomas are primary tumors that are thought to derive from cancer stem cells (CSC) sharing with neural stem cells (NSC) the capacity of cell renewal and multipotency [1]. Glioma includes morphologically distinct cancers such as astrocytoma, ependymoma and oligodendroglioma in which astrocytoma is the most common form [2]. Glioma accounts for about 30% of all primary brain tumors and 80% of intracranial malignancies [3,4]. Historically, based on the degree of malignancy, gliomas are roughly classified into four grades, I to IV [5]. Compared with grades I-II, grades III-IV have a higher degree of malignancy and a faster progression time, suggestive of a high-grade malignant tumor. About half of the gliomas are glioblastoma (GBM, WHO class IV astrocytoma), the most common primary tumor in the adult brain [5], with a median survival of only 15 months. The overall 5-year survival rate is still below 5% [6]. Recently, the classification of gliomas was updated by the 2016 World Health Organization Classification of Tumors of the Central Nervous System using molecular parameters in addition to histology [7,8]. Although the qualitative and localized diagnosis of glioma is very mature, the current comprehensive treatments of glioma, including surgery, radiotherapy and chemotherapy, only prolong the survival of patients for several months [9,10]. Therefore, it remains critical to find new treatments for glioma.

The failure of GBM treatment partially results from the high degree of molecular and functional heterogeneity between tumors [11,12,13]. GBM and lower grade gliomas contain a subpopulation of cells with stem cell properties that are functionally defined as glioblastoma stem cells (GSCs) [14]. The abnormal differentiation potential of GSCs is an important feature of GBM [15]. Therefore, strategies to induce GSC differentiation have been applied to GBM. Park et al. identified a subset of GSCs that express high levels of the proneural transcription factor ASCL1 (Achaete-scute complex-like 1) which exhibits a latent capacity for terminal neuronal differentiation in response to Notch inhibition, effectively attenuating tumorigenicity [16]. It was also reported that retinoic acid, bone morphogenetic proteins or histone deacetylase inhibitors could induce GSC differentiation into a glial cell fate and inhibit tumor exponential growth [17,18,19]. In the preclinical and clinical settings, however, the efficacy of the differentiation therapy appears to be limited [20], fostering aggressive investigations into alternative therapeutic paradigms. 

In addition to the abnormal differentiation potential of GSCs, the deadly nature of glioma also originates from its invasive proliferation of brain tissue. Therefore, much of the current molecular research focuses on how to inhibit glioma cell proliferation. For example, overexpression of Pax6 [21], Pten [22] or P53 [23] in glioma cells was shown to inhibit their proliferation or induce their apoptosis. Recently, reprogramming technologies, including somatic cell nuclear transfer (SCNT), induced pluripotent stem cells (iPSCs) and direct lineage reprogramming, may also pave the way for potential glioma treatment by altering cell plasticity. By direct neuronal reprogramming, non-neuronal somatic cells can be converted into neurons, the terminally differentiated cells, which do not undergo a pluripotent phase during lineage conversion, thus possibly avoiding teratoma formation [24,25,26]. It has been reported that fibroblasts and astrocytes can be successfully reprogrammed into neurons introduction of specific neurogenic transcription factors [27,28,29]. Because the reprogrammed neurons are terminally differentiated cells that do not proliferate, direct neuronal reprogramming of glioma cells could potentially represent an effective treatment for brain tumors by blocking their aggressive proliferation. 

Recent study has showed that cultured human glioma cells could be converted into neuron-like cells by overexpression of three neurogenic transcription factors ASCL1, BRN2 and NGN2 (Neurogenin-2) [30]. Our previous study also identified that NGN2 and SOX11 (sex-determining region Y-box 11) could cooperatively reprogram malignant glioma cells into postmitotic neuron-like cells, leading to significant inhibition of tumor growth [31]. However, it is unknown whether a single transcription factor is sufficient to induce the human glioma cells reprogramming into postmitotic neurons. As an evolutionarily conserved basic-helix-loop-helix (bHLH) transcription factor, ASCL1 is an important regulator of neurogenesis in both the peripheral nervous system (PNS) and the central nervous system (CNS) [32,33,34]. Ectopic expression of ASCL1 in somatic non-neural cells was shown to induce neuronal fate and resulted in cell-cycle exit [28,35]. 

In the present study, we screened a series of transcription factors and found that ASCL1 alone could rapidly, efficiently and directly reprogram human glioma cells into non-proliferating neurons. This glioma-to-neuron conversion was completed without passing through an intermediate pluripotent state and led to significant inhibition of tumor growth.

## 2. Materials and Methods

### 2.1. Animals

The immunodeficient NOD-SCID gamma (NSG) mice used in our study were all from Shanghai Ling chang Biotech Co., Ltd. They were placed under a controlled temperature with a 12 h light/dark cycle and were provided with sufficient food and drinking water. In this study, the National Institutes of Health Laboratory Animal Care and Use Guidelines were carefully followed and all the experimental procedures and protocols were approved by the Animal Ethics Committee of the Second Military Medical University.

### 2.2. Cell Culture

HEK-293T cells, U251 cells and U87 cells were cultured in DMEM medium containing 10% fetal bovine serum and 1% penicillin/streptomycin. HEK-293T cells were planted in a 10-cm dish and used for virus packaging. The human glioma cells (U251) were seeded on glass slides coated with or without gelatin/matrigel and cultured in culture vessels. For neuronal reprogramming, U251 cell culture medium was switched to a neuronal induction medium on the second day after infection with virus. The components of the neuronal induction medium included DMEM:F12:Neurobasal (2:2:1), 0.8% N-2 (Invitrogen, Carlsbad, CA, USA) and 0.4% B-27 (Invitrogen, Carlsbad, CA, USA), and forskolin (FSK, 10 μM) and dorsomorphin (DM, 1 μM). The induction medium was changed every other day during the lineage reprogramming process.

### 2.3. Plasmid Construction and Lentiviral Packaging

Plasmid construction was carried out with reference to our previous protocol [31]. Briefly, the mouse or human-derived candidate genes were amplified by PCR and sub-cloned into a lentiviral vector in which gene expression was regulated by a human glial fibrillary acidic protein (hGFAP) promoter. GFP co-expressed in the vector was used to visualize the cells infected with virus. The lentiviral vector and the packaging plasmid (pMDL, VSV-G, pREV) were transiently transfected into HEK-293T cells and the medium was changed 12 h after transfection. We collected the replication-deficient lentivirus at 24 and 48 h. For neuronal reprogramming, the glioma cells were infected with the virus for eight–12 h and switched to neuronal induction medium. The medium was changed every other day.

### 2.4. Data from GEO and TCGA 

The microarray dataset (GSE15824) was obtained from the public database Gene Expression Omnibus (GEO) [36,37]. We selected eight human astrocytoma samples and three normal human astrocyte samples for comparative analysis. The data for the analysis of gene expression and survival curves were obtained from The Cancer Genome Atlas (TCGA) [38], in which 676 glioma samples were selected for analysis. All the data were analyzed via R (version 3.5.0). 

### 2.5. Immunofluorescent and Histological Staining

For immunocytochemistry, cells cultured on the paddles were fixed with 4% paraformaldehyde (PFA) in PBS for 20 min at room temperature, blocked with 0.2% Triton X-100 and 3% BSA in 1×PBS for one h, and then incubated overnight at 4 °C in appropriate concentrations of the primary antibodies (Supplementary Table S1). After washing with 1×PBS, cells were incubated with the secondary antibodies conjugated to Alexa Fluor 488, 594 or 647 (Jackson ImmunoResearch, West Grove, PA, USA) for two h at room temperature for indirect fluorescence. For immunohistochemistry, brain tissues were fixed by intracardiac perfusion with 4% PFA in PBS. Sections (20 μm) were processed for immunofluorescence as described in immunocytochemistry. Following immunocytochemistry or immunohistochemistry, nuclei were counterstained with Hoechst 33342 (Hst) for 15 min. In addition, for in vivo quantification, tumor sizes were quantified from 10 representative 40-μm-thick serial coronal brain sections that were stained with hematoxylin and eosin (HE). Images were captured with a Leica confocal microscope, or a Nikon E600FN microscope.

### 2.6. Immunoblots

Western blot analysis was performed as previously described [39]. The total proteins extracted from cultured U251 cells were electrophoresed on a 10% SDS-PAGE gel, transferring onto nitrocellulose membranes. After blocking with 5% non-fat milk for two h, the membranes were incubated with specific primary antibodies against ASCL1 (BD Biosciences, San Jose, CA, USA; mouse, 1:200) and β-actin (Proteintech, Chicago, IL, USA; mouse, 1:5000), followed by incubation with the secondary antibodies for two h at room temperature. The integrated optical density (IOD) was calculated by using BIO-RAD Molecular Imager.

### 2.7. Cell Proliferation Assays

BrdU (5-bromo-2-deoxyuridine) incorporation assay was performed to determine cell proliferation. The proliferating cells were labeled by incubation with 10 mM BrdU (Sigma B5002). The BrdU incorporation was detected by fluorescent staining using an anti-BrdU antibody (ThermoFisher, Waltham, MA, USA; MA1-82088). In addition, the proliferating cells were also labeled directly by staining with antibodies against Ki67 (Abcam, Cambridge, UK; ab65260).

Time-course cell counting was used to assess glioma cell growth. Briefly, glioma cells were seeded in a 24-well plate (4 × 10^4^ cells/well). The cell numbers were counted at 0, 1, 3, 5, 7 and 14 days after infection with lentivirus.

### 2.8. Intracranial Tumor Growth Assay

To investigate the effects of ASCL1-mediated neuronal reprogramming on tumor growth in vivo, human U251 glioma cells were infected with lentivirus expressing either ASCL1 or control GFP. Three days later, the infected U251 cells (5 × 10^5^ cells in 2 μL) were stereotactically injected into the striatum of NSG mice according to the injection coordinates as follows: +1.0 mm AP; +2.0 mm ML and −3.0 mm DV. The brain tissues were fixed by intracardiac perfusion with 4% PFA at 3 weeks after transplantation, followed by immunohistochemical analysis and HE staining.

### 2.9. Quantitative and Statistical Analysis

Quantitative data were from 20 randomly selected fields derived from at least three independent experiments. The total number of cells was not less than 500 cells. All experimental data are expressed as mean ± SD. The statistical analysis was performed using the unpaired Student’s *t*-test. *P* < 0.05 was considered to be statistically different.

## 3. Results

### 3.1. Screening of A Single Transcription Factor to Convert Human Malignant Glioma Cells into Neuron-Like Cells

**Figure 1:**
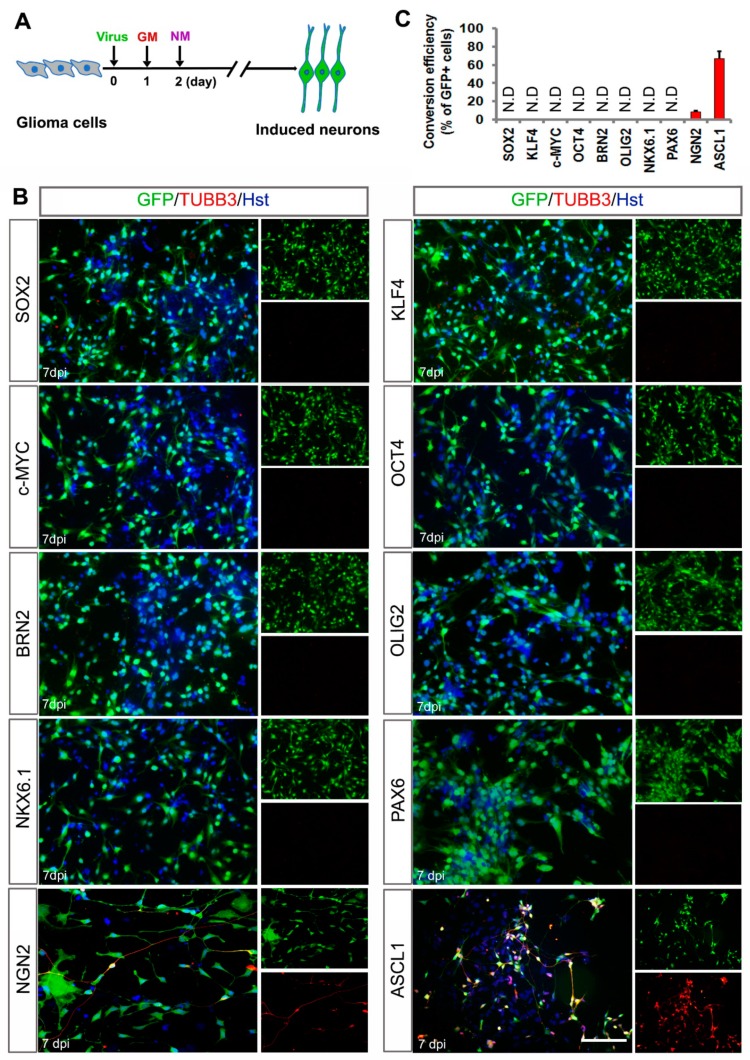
Identifying a single transcription factor to induce neuronal fate on human glioma cells. (**A**) Experimental schemes of transcription factor-mediated neuronal reprogramming. GM, growth medium; NM, neuronal induction medium. (**B**) Immunocytochemical analysis of tubulin beta-3 (TUBB3)-positive neuron-like cells seven days post infection of U251 cells with virus expressing the indicated transcription factors. Virus-transduced cells are indicated by the co-expressed GFP marker. (**C**) Quantification of U251 cell-converted TUBB3+ neuron-like cells. Conversion efficiency was normalized to GFP-positive cells. N.D, not detected. Scales = 100 μm.

Previously, we and others have reported that the combined transcription factors (BRN2/ASCL1/ MYT1L or NGN2/SOX11) could reprogram human malignant glioma cells into terminally differentiated neuron-like cells and successfully impede brain tumor development [30,31]. Here, our study focused on identifying a single transcription factor that is sufficient to induce neuronal reprogramming of human malignant glioma cells. Based on the knowledge of their roles in NSCs and/or neurogenesis, 11 genes (*ASCL1*, *PAX6*, *NKX6.1*, *BRN2*, *NGN2*, *SOX11*, *OLIG2*, *OCT4*, *c-MYC*, *KLF4* and *SOX2*) were selected as candidates. A lentiviral gene delivery system was used to target human glioma cells. For screening, the cultured U251 cells were individually infected with the lentivirus expressing these transcription factor candidates under the regulation of an *hGFAP* promoter (Supplementary Figure S1). At 7 days postinfection (dpi), neuronal reprogramming was examined by staining for the expression of TUBB3, a pan-neuronal marker. Figure 1 showed that overexpression of ASCL1 resulted in extensive production of TUBB3-positive cells in the cultures, whereas the expression of TUBB3 was undetected in the cultures infected with *PAX6*, *NKX6.1*, *BRN2*, *SOX11*, *OLIG2*, *OCT4*, *c-MYC*, *KLF4* or *SOX2*. Of note, only a few TUBB3-positive cells were induced from NGN2-infected U251 cells, consistent with our previous study [31].

**Figure 2:**
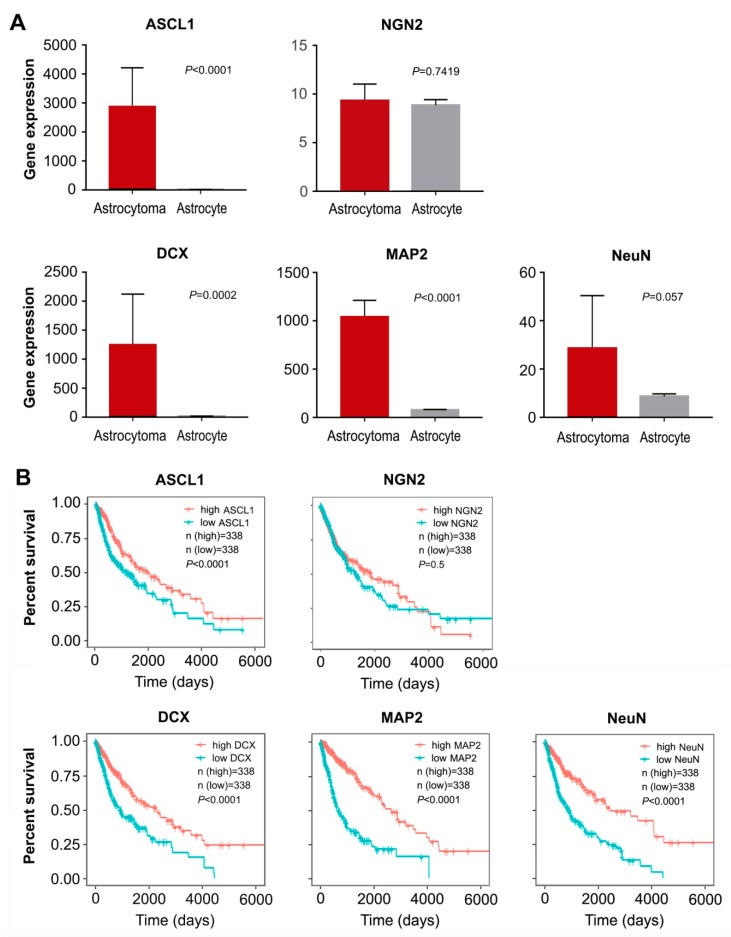
Data analysis from GEO and TCGA. (**A**) The differential expression of transcription factors (ASCL1 and NGN2) and neuronal markers (DCX, MAP2 and NeuN) in human astrocytoma and astrocytes from the data obtained from GEO (mean ± SD, Student’s *t*-test). (**B**) Analysis of gene expression and survival curves based on the data of 676 glioma samples obtained from TCGA.

Additionally, we performed gene differential expression analysis on human astrocytoma and astrocytes from the data obtained from the public database Gene Expression Omnibus (GEO) [37]. As shown in Figure 2A, high expression level of ASCL1 was observed in human astrocytomas, compared with human normal astrocytes. However, no significant difference was detected in the expression of NGN2 between astrocytomas and astrocytes (Figure 2A). Interestingly, we also found that some neuronal markers such as DCX, MAP2 and NeuN were highly expressed in astrocytomas (Figure 2A). Furthermore, analysis of gene expression and survival curves was performed on the data of 676 glioma samples obtained from The Cancer Genome Atlas (TCGA) [38], showing that the patients with high expression of transcription factor ASCL1 but not NGN2 had a better survival (Figure 2B). Similarly, the high expression of neuronal markers DCX, MAP2 and NeuN were also mirrored by a better survival (Figure 2B). Taken together, all these results suggested that the single transcription factor ASCL1 may be sufficient to reprogram glioma cells into neurons, representing a feasible strategy for human malignant glioma treatment.

### 3.2. ASCL1-Mediated highly Efficient Conversion of Human Malignant Glioma Cells into Neurons 

**Figure 3:**
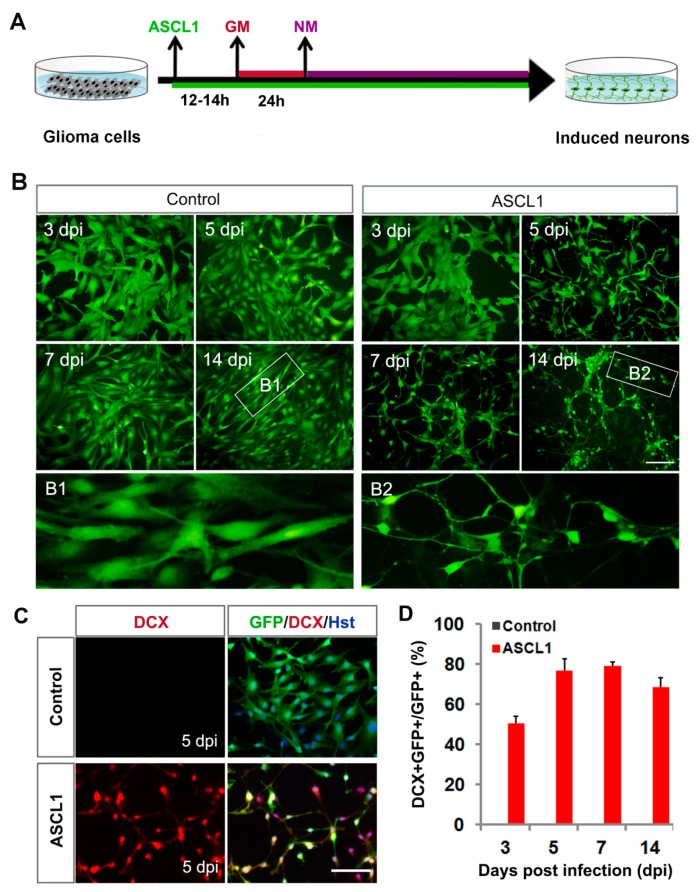
Fate change of human glioma cells induced by ASCL1. (**A**) Experimental schemes of ASCL1-mediated lineage reprogramming. GM, growth medium; NM, neuronal induction medium. (**B**) Ectopic expression of ASCL1 resulted in rapid morphological changes of U251 cells. GFP-expressing virus was used as a control. Higher magnification views of the boxed regions are also shown. (**C**) ASCL1-converted cells expressed immature neuronal marker DCX. (**D**) Quantitative analysis of induced DCX-positive cells. Scales: 100 μm (**B**,**C**).

We next investigated the roles of ASCL1 in inducing neuronal reprogramming of human malignant glioma cells. The cultured U251 cells were infected with ASCL1-expressing lentivirus. Based on the co-expressed GFP and nuclear marker Hoechst (Hst), the infection efficiency was estimated to be more than 97%, and there was no significant difference between ASCL1 and the control GFP group (Supplementary Figure S2A,B). Western blot analysis confirmed that the ASCL1 protein was indeed ectopically expressed in the infected U251 cells (Supplementary Figure S2C). As shown in Figure 3A, after infection of U251 cells with ASCL1-expressing lentivirus, the serum containing culture medium was switched to forskolin (FSK)- and dorsomorphin (DM)-supplemented neuronal induction medium. A time-course morphological analysis showed that forced expression of ASCL1 induced U251 cells to change morphology as early as five days post infection (dpi) (Figure 3B). They rapidly lost their polygonal or spindle morphology and adopted a neuronal appearance with long neurites. At seven and 14 dpi, they acquired a more complex morphology with multiple neuron-like processes. In sharp contrast, no significant change of cell morphology was observed in the control virus-infected group (Figure 3B). Consistent with the morphological observation, immnuocytochemistry revealed that doublecortin (DCX), a microtubule-associated protein that is broadly expressed in neuroblasts and immature neurons [40,41], was detected in the ASCL1 virus-treated culture with a peak level at seven dpi, whereas the control virus treatment did not result in significant expression of DCX (Figure 3C,D). These DCX-positive cells showed typical morphology for neuroblasts/immature neurons with bipolar or multipolar processes and were labelled by GFP, suggestive of an origin of virus-transduced cells (Figure 3 C).

**Figure 4:**
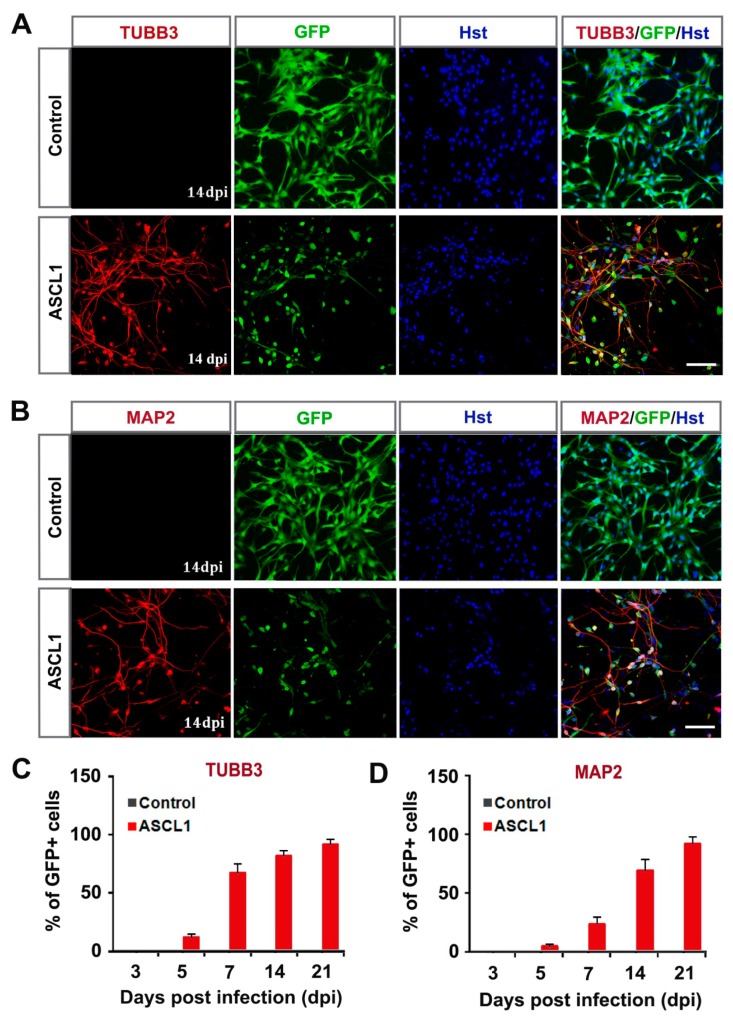
Time-course analysis of ASCL1-mediated neuronal reprogramming of human glioma cells. (**A**,**B**) Immunostaining of neuronal markers TUBB3 and MAP2 in ASCL1converted cells at 14 dpi. (**C**,**D**) Quantification of neuronal marker expressing in ASCL1-infected U251 cells during the indicated course (n = 20 random fields from triplicated samples). Scales: 100 μm (**A**,**B**).

The neuronal fate reprogramming was further validated by immunocytochemical analysis of the expression of pan-neuronal markers, TUBB3 and MAP2. As shown in Figure 4A,B, both the early neuronal marker TUBB3 and the mature neuronal marker MAP2 could be detected in the ASCL1-induced cells from U251 cells. However, neither TUBB3 nor MAP2 signal was observed in culture infected with control GFP-expressing virus (Figure 4A,B). A time-course analysis of TUBB3 indicated that it was expressed in ASCL1-infected cells (indicated by GFP fluorescence) as early as five dpi, and sharply increased by seven dpi (Figure 4C). At 21 dpi, 94.3% of ASCL1-infected U251 cells were converted into TUBB3-positive neuron-like cells (Figure 4C). A similar expression pattern of MAP2 was observed during the ASCL1-mediated neuronal reprogramming process (Figure 4D). At 21 dpi, more than 94% of ASCL1-infected U251 cells were reprogrammed into MAP2-positive neuron-like cells (Figure 4D). To determine whether the ASCL1-mediated neuronal reprogramming was applicable to other human glioma cell line, U87 cells were also tested in our study. Consistent with U251, the time-course immunocytochemical analysis with neuronal markers DCX, TUBB3 and MAP2, showed that ASCL1 alone could also rapidly and efficiently reprogram U87 human glioma cells into neuron-like cells (Supplementary Figure S3).

### 3.3. Subtype Specification of Glioma Cell-Derived Neurons

**Figure 5:**
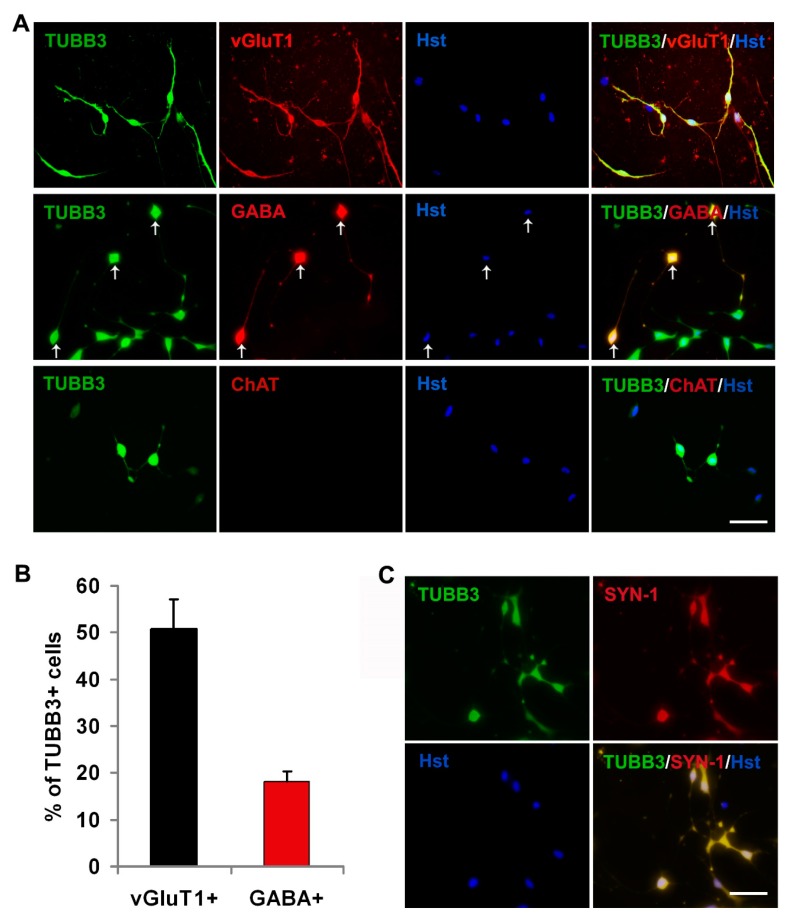
Characterization of glioma cell-derived neurons. (**A**) Cellular identity analysis of ASCL1-induced neurons using specific neuronal markers vGluT1, GABA and ChAT at 21 dpi. (**B**) Quantification of neuronal subtypes (n = 20 random fields from triplicate samples). (**C**) Expression of presynapic markers synapsin-1 (SYN-1) at 21 dpi. Scales: 50 μm (**A**,**C**).

During CNS development, ASCL1 is expressed in progenitor cells committed to the generation of GABAergic interneurons [42,43]. For lineage reprogramming, ASCL1 converts astrocytes from different origins into neurons adopting mostly a GABAergic phenotype or a mix of glutamatergic and GABAergic phenotypes [29,43,44,45]. Therefore, immunocytochemical analysis was performed to examine the subtype of glioma cell-converted neurons using specific markers, including vGluT1 (excitatory interneuron marker), GABA (inhibitory interneuron marker) and ChAT (motor neuron marker). As shown in Figure 5A, both vGluT1 and GABA -positive neurons could be observed in the ASCL1-mediated glioma cell-to-neuron system, while no ChAT signal was detected in the culture, suggesting that ASCL1 reprogrammed glioma cells into inhibitory/excitatory interneurons but not motor neurons. Quantitative analysis of neuronal subtypes at 21 dpi revealed that >50% of ASCL1-induced TUBB3-labeled neurons were positive for vGluT1, whereas <20% of them were GABA-positive (Figure 5B). These data indicated that ectopic expression of ASCL1 instructs glioma cells reprogramming into a hybrid of interneurons, mainly excitatory neurons. Additionally, confocal analysis by immunostaining showed that the presynaptic neuronal marker synapsin-1 (SYN-1) could be detected in discrete puncta in the glioma cell-converted neurons, suggestive of synapse-forming neurons (Figure 5C).

### 3.4. ASCL1-Induced Neuronal Reprogramming Results in Cell Cycle Exit and Inhibits Glioma Growth

**Figure 6:**
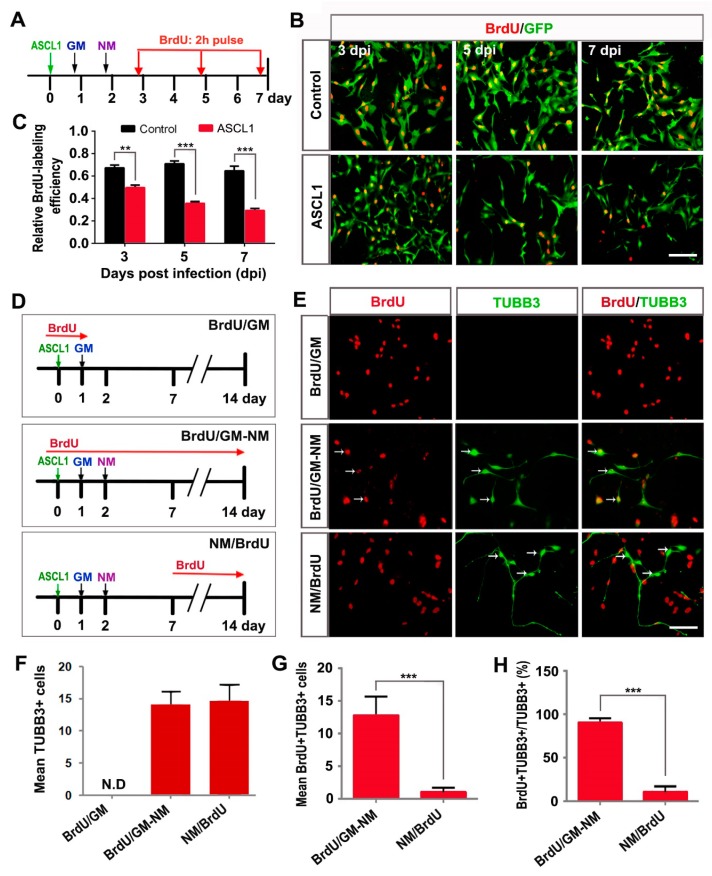
ASCL1-induced neuronal reprogramming results in cell cycle exit. (**A**) The experimental scheme for data presented in panels B and C. After virus infection, U251 cells were incubated with BrdU for two h before immunocytochemistry at the indicated time points. GM, growth medium; NM, neuronal induction medium. (**B**,**C**) Time-course analysis of proliferating cells after infection of U251 cells with ASCL1 or control GFP virus (n = 20 randomly selected fields from three independent experiments; ** *P* < 0.01, *** *P* < 0.001 by Student’s *t*-test). (**D**) The experimental scheme for data presented in panels E-H. U251 cells were infected with ASCL1-expressing virus and treated with BrdU for the indicated periods of time. (**E**–**H**) Quantitative analysis of reprogrammed cells and cell proliferation (n = 20 randomly selected fields from triplicate samples; *** *P* < 0.001 by Student’s *t*-test). ASCL1 induced no neuron-like cells under the growth medium (GM) condition. The neuronal survival was not affected by continuous incubation with BrdU (BrdU/GM-NM). ASCL1-induced neuronal reprogramming led to rapid cell cycle arrest (NM/BrdU). The “mean number of cells” refers to the average number of cells per field of view. Arrows indicate BrdU^+^TUBB3^+^ cells. N.D, not detected. Scales: 100 μm (**B**,**E**).

Because neuron is a population of postmitotic cells, we further determined whether the aggressive proliferation of glioma cells could be inhibited by the ASCL1-mediated neuronal reprogramming. After infection with control GFP or ASCL1 virus, the U251 cells were switched to neuronal induction medium. To label the proliferating cells, the cultures were treated with BrdU (5-bromodeoxyuridine, 10 μM) for two h before immunocytochemical analysis at three, five or seven dpi (Figure 6A). At three dpi, about 70% of control GFP-infected cells and 50% of ASCL1-infected cells were labeled by BrdU (Figure 6B,C). Compared with the control group, the time-course quantitative analysis showed that forced expression of glioma cells with ASCL1 lentivirus resulted in a dramatic decrease in the number of BrdU-labeled cells (Figure 6B,C), suggesting that ASCL1-mediated neuronal reprogramming might lead to cell cycle exit of glioma cells (Figure 6D). Continuous BrdU labeling was also performed to examine the proliferative activity of glioma cell-converted neurons. As shown in Figure 6D–H, when BrdU was administrated prior to the ASCL1 virus infection, most of the non-converted TUBB3-negative cells in BrdU/GM group and the converted TUBB3-positive neurons were labeled by BrdU. However, when BrdU was administrated at seven dpi, only a few ASCL1-converted TUBB3-positive neurons were labeled by BrdU (Figure 6D–H). All these findings suggested that ASCL1 could reprogram human malignant glioma cells into terminally differentiated neurons and resulted in cell cycle arrest.

**Figure 7:**
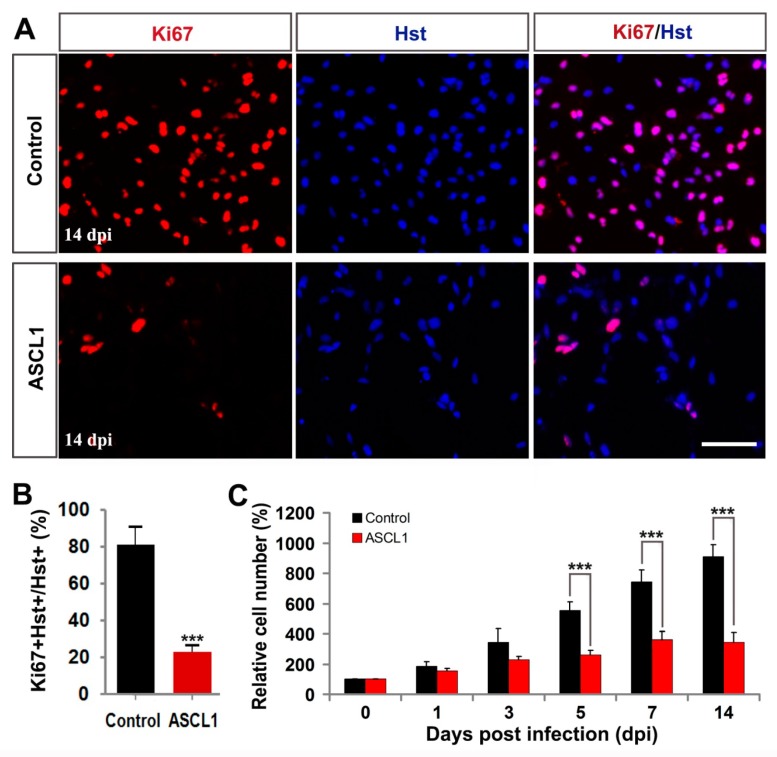
ASCL1-mediated neuronal reprogramming significantly inhibited glioma cell proliferation. (**A**,**B**) Immunocytochemical analysis of the proliferation of U251 cells by Ki67 staining at 14 dpi (n = 20 randomly selected fields from triplicate samples). (**C**) Time-course cell counting at zero, one, three, five, seven, and 14 dpi. *** *P* <0.001 by Student’s *t*-test. Scales = 100 μm.

To determine whether ASCL1-induced cell cycle exit contributed to inhibiting the glioma cell growth, immunocytochemistry and quantitative analysis were performed. As an endogenous marker for proliferating cells, Ki67 staining was used to assess the growth of glioma cells after infection with control GFP or ASCL1 lentivirus. At 14 dpi, when a majority of glioma cells had been reprogrammed into neurons by ASCL1 (Figure 4), immunocytochemical analysis showed that 81.4% of glioma cells were positive for Ki67 in the control group, whereas only 23.1% of them were Ki67-positive in the ASCL1 group (Figure 7A,B). In the culture treated with ASCL1-expressing lentivirus, most of ASCL1-infected (GFP^+^) cells acquired a neuron-like morphology (Supplementary Figure S4A). Of note, only 18.8% of the ASCL1-expressing (GFP^+^) cells were labeled by Ki67, while 81.2% of the Ki67-positive cells were detected in the non-infected (GFP^-^) glioma cells (Supplementary Figure S4A,B). As shown in Figure 7C, a time-course cell counting indicated that a continued aggressive growth was observed in the glioma cells infected with control GFP-expressing virus. After infection glioma cells with ASCL1-expressing virus, however, their growth reached a plateau at seven dpi and no significant expansion was detected thereafter (Figure 7C). Collectively, these data suggested that the exponential growth of human malignant glioma cells could be inhibited by the ASCL1-induced neuronal reprogramming.

### 3.5. ASCL1-Mediated Neuronal Reprogramming Inhibits the Tumorigenic Potential of Glioma Cells In Vivo

**Figure 8:**
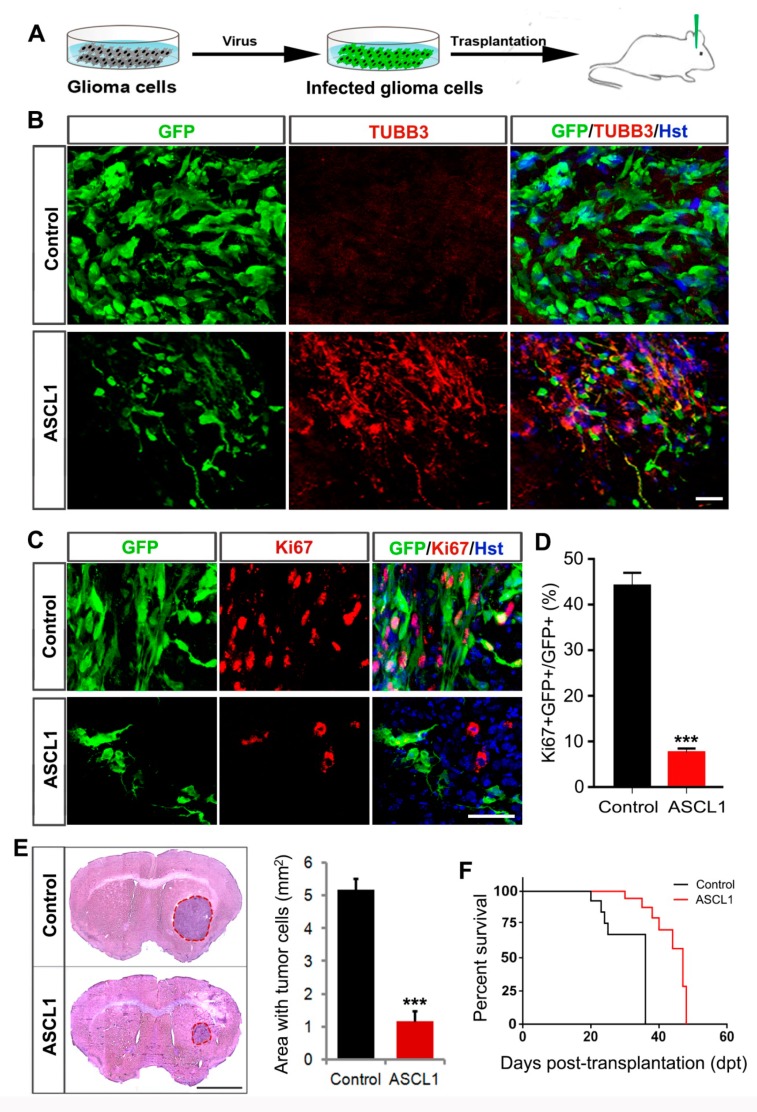
Effects of ASCL1-mediated neuronal reprogramming on the tumorigenicity of glioma cells in vivo. (**A**) The experimental scheme for data presented in panels B–D. (**B**) Representative images showing glioma cell-derived neurons in the brain three weeks after transplantation with ASCL1 but not control virus-infected U251 cells. (**C**,**D**) Three weeks after transplantation into the mouse brain, immunohistochemical analysis was performed on the proliferation of ASCL1 or control virus-infected glioma cells. Quantification data were from 20 randomly selected fields from triplicate samples. (**E**) Histological analysis of tumor formation in mice transplanted with U251 cells that were infected with control or ASCL1 virus. Tumor mass (outlined by dashed red lines) was quantified from HE-stained coronal brain sections three weeks after transplantation (n = 4 for each group; *** *P* < 0.001 by Student’s t-test). (**F**) Kaplan–Meier survival curve of mice transplanted with U251 cells that were infected with the indicated virus (n = 11 for each group; P = 0.001 by log-rank test). Scales = 50 μm (**B**,**C**), 1 mm (**E**).

We next performed orthotopic cell transplantation experiments to determine whether the ASCL1-mediated neuronal reprogramming could inhibit the tumorigenic potential of human glioma cells in vivo (Figure 8A). In vitro, cultured human U251 cells were infected with ASCL1 or control GFP-expressing lentivirus. Three days later, these virus-infected human U251 cells were stereotactically injected into the striatum of NSG mice. The co-expressed GFP was used to trace the engrafted human glioma cells infected with virus. At three weeks post transplantation, marked GFP-labeled tumor masses were readily detectable in the brains transplanted with the control GFP but not ASCL1-infected U251 cells (data not shown), suggestive of the reduced tumorigenic activity of ASCL1-expressing human glioma cells in vivo. As shown in Figure 8B, a majority of engrafted ASCL1-infected human glioma cells acquired neuron-like morphology and expressed the pan-neuronal marker TUBB3. However, no TUBB3-positive neuron-like cells was observed in the mouse brain injected with control GFP-infected human glioma cells (Figure 8B). 

To determine whether the ASCL1-mediated in vivo neuronal reprogramming inhibited the aggressive proliferation of transplanted human glioma cells, immunohistochemical analysis was performed by Ki67 staining. Figure 8C,D showed that around 45% of control GFP-expressing human glioma cells were stained positive for Ki67, whereas less than 8% of ASCL1-expressing human glioma cells were Ki67-positive. Three weeks after transplantation, furthermore, the tumor burden was analyzed by HE staining. As shown in Figure 8E, the ASCL1-infected U251 glioma cells resulted in a much smaller tumor in brain than that were infected with control GFP virus. Accordingly, the mice transplanted with ASCL1-infected U251 glioma cells survived significantly longer than those engrafted with control GFP-infected U251 glioma cells (Figure 8F). All these findings indicated that the single transcription factor ASCL1 could also reprogram human glioma cells into terminally differentiated neurons in vivo, which contributed to blocking the aggressive proliferation of human glioma cells and inhibiting their tumorigenicity in the brain.

## 4. Discussion

Although the direct neuronal reprogramming has been well documented as an important technology for regenerative medicine, little is known about its application for tumor treatment. Based on the fact that neurons are terminally differentiated, reprogramming of cancer cells into neurons might represent a potential strategy to inhibit their aggressive proliferation [30,31,46]. Although the transcription factor ASCL1 is required for GSCs to undergo neuronal lineage differentiation [16], it remains unknown whether ASCL1 can induce neuronal reprogramming of glioma cells. Here, we showed that ASCL1 alone could rapidly, efficiently, and directly reprogram human glioma cells into non-proliferating neurons, resulting in significant inhibition of tumor growth.

Both ASCL1 and NGN2 are evolutionarily conserved basic-helix-loop-helix (bHLH) transcription factors. They have been widely described in the developing CNS as master regulators of neural fate and specification of neuronal identities. During brain development, ASCL1 and NGN2 instruct stem and progenitor cells in diverse brain regions differentiation into GABAergic and glutamatergic neurons, respectively [33,47]. Besides the pivotal roles in CNS neurogenesis, ASCL1 and NGN2 are also pioneering transcription factors that trigger neuronal reprogramming [29,44,45,48]. By screening, we here found that ASCL1 or NGN2 alone is sufficient to convert human glioma cells into neuron-like cells. Of note, NGN2 induced human glioma cell-to-neuron conversion with a low conversion rate. However, ASCL1 could rapidly and efficiently reprogram human glioma cells into neuron-like cells. The ASCL1-induced neuron-like cells were detected as early as five dpi. By 14 dpi, more than 94% human glioma cells had been converted into MAP2-positive mature neurons. 

In spite of the well-defined fact that ASCL1 instructs progenitors to generate GABAergic interneurons in the neocortex, this proneural factor cannot be simply regarded as a GABAergic determinant. In fact, ASCL1 was shown to drive the acquisition of diverse neuronal subtypes throughout the CNS, such as glutamatergic neurons in the retina, noradrenergic neurons in the locus coeruleus, and cholinergic neurons in the basal ganglia [43,49]. For direct neuronal reprogramming, ASCL1 converted postnatal cerebral cortex and cerebellum astrocytes into induced neurons adopting mostly a GABAergic phenotype [29,44,45]. However, when overexpressed in midbrain astrocytes, ASCL1 induced a mix of glutamatergic and GABAergic neurons [50]. In our study, ectopic expression of ASCL1 in human glioma cells instructed them reprogramming into a hybrid of neurons, including glutamatergic and GABAergic interneurons. These results suggested that the neuronal subtype specification by ASCL1-mediated lineage reprogramming might be dependent on the context of donor cells. For example, the donor cells were shown to retain a molecular memory of their tissue origin and affect the phenotype of induced neurons [51,52,53]. In addition, the acquisition of alternative neuronal phenotypes might also be regulated by the post-transcriptional modifications of ASCL1 [54,55].

The malignant gliomas are essentially incurable mainly due to their invasive proliferation. Physiologically, the oscillatory expression pattern of ASCL1 in progenitor cells of the ventral telencephalon contributes to their proliferation and multipotency, whereas the sustained expression of ASCL1 activates genes driving cell cycle exit and neuronal differentiation [56,57]. In our study, ASCL1-induced neuronal reprogramming of human malignant glioma cells resulted in cell cycle exit and blocked their aggressive proliferation. When human glioma cells infected with ASCL1-expressing lentivirus were transplanted into the mouse striatum, they could also be converted into terminally differentiated neurons in vivo. Importantly, this lineage reprogramming significantly inhibited tumor growth and extended their lifespan. Of note, data analysis of gene expression and survival curves of 676 glioma samples obtained from TCGA [38] showed that the patients with high expression of transcription factor ASCL1 and neuronal markers had a better survival.

In summary, this proof-of-concept study indicated that the single transcription factor ASCL1 was sufficient to high-efficiently reprogram human glioma cells into non-proliferating neurons and restricted tumor growth, suggestive of a promising potential for brain tumor treatment. However, further studies will be needed to increase the conversion efficiency and glioma targeting specificity of viral transduction. For example, multiple site injections and continuous infusion of larger volume using a cannula may be effective for in vivo tumor treatment. To specifically target tumor cells but not surrounding normal cells, a different virus-mediated gene delivery system with glioma cell type-specific promoter may also be identified. 

## Supplementary Materials

Supplementary materials are available online at https://www.mdpi.com/2073-4409/8/6/571/s1.

## References

[1] Dell’Albani P. (2008). Stem cell markers in gliomas. Neurochem. Res..

[2] Sukhdeo K., Hambardzumyan D., Rich J.N. (2011). Glioma development: Where did it all go wrong?. Cell.

[3] Hadziahmetovic M., Shirai K., Chakravarti A. (2011). Recent advancements in multimodality treatment of gliomas. Future Oncol..

[4] Chen J., McKay R.M., Parada L.F. (2012). Malignant glioma: Lessons from genomics, mouse models, and stem cells. Cell.

[5] Louis D.N., Ohgaki H., Wiestler O.D., Cavenee W.K., Burger P.C., Jouvet A., Scheithauer B.W., Kleihues P. (2007). The 2007 WHO classification of tumours of the central nervous system. Acta Neuropathol..

[6] Bleeker F.E., Molenaar R.J., Leenstra S. (2012). Recent advances in the molecular understanding of glioblastoma. J. Neurooncol..

[7] Louis D.N., Perry A., Reifenberger G., Von Deimling A., Figarella-Branger D., Cavenee W.K., Ohgaki H., Wiestler O.D., Kleihues P., Ellison D.W. (2016). The 2016 World Health Organization Classification of Tumors of the Central Nervous System: A summary. Acta Neuropathol..

[8] Malzkorn B., Reifenberger G. (2016). Practical implications of integrated glioma classification according to the World Health Organization classification of tumors of the central nervous system 2016. Curr. Opin. Oncol..

[9] Emmanuel C., Lawson T., Lelotte J., Fomekong E., Vaz G., Renard L., Whenham N., Raftopoulos C. (2018). Long-term survival after glioblastoma resection: Hope despite poor prognosis factors. J. Neurosurg. Sci..

[10] Walid M.S. (2008). Prognostic factors for long-term survival after glioblastoma. Perm. J..

[11] Brennan C.W., Verhaak R.G., McKenna A., Campos B., Noushmehr H., Salama S.R., Zheng S., Chakravarty D., Sanborn J.Z., Berman S.H. (2013). The somatic genomic landscape of glioblastoma. Cell.

[12] Meyer M., Reimand J., Lan X., Head R., Zhu X., Kushida M., Bayani J., Pressey J.C., Lionel A.C., Clarke I.D. (2015). Single cell-derived clonal analysis of human glioblastoma links functional and genomic heterogeneity. Proc. Natl. Acad. Sci. USA.

[13] Patel A.P., Tirosh I., Trombetta J.J., Shalek A.K., Gillespie S.M., Wakimoto H., Cahill D.P., Nahed B.V., Curry W.T., Martuza R.L. (2014). Single-cell RNA-seq highlights intratumoral heterogeneity in primary glioblastoma. Science.

[14] Singh S.K., Hawkins C., Clarke I.D., Squire J.A., Bayani J., Hide T., Henkelman R.M., Cusimano M.D., Dirks P.B. (2004). Identification of human brain tumour initiating cells. Nature.

[15] Chen J., Li Y., Yu T.S., McKay R.M., Burns D.K., Kernie S.G., Parada L.F. (2012). A restricted cell population propagates glioblastoma growth after chemotherapy. Nature.

[16] Park N.I., Guilhamon P., Desai K., McAdam R.F., Langille E., O’Connor M., Lan X., Whetstone H., Coutinho F.J., Vanner R.J. (2017). ASCL1 Reorganizes Chromatin to Direct Neuronal Fate and Suppress Tumorigenicity of Glioblastoma Stem Cells. Cell Stem Cell.

[17] Ying M., Wang S., Sang Y., Sun P., Lal B., Goodwin C.R., Guerrero-Cazares H., Quinones-Hinojosa A., Laterra J., Xia S. (2011). Regulation of glioblastoma stem cells by retinoic acid: Role for Notch pathway inhibition. Oncogene.

[18] Massard C., Deutsch E., Soria J.C. (2006). Tumour stem cell-targeted treatment: Elimination or differentiation. Ann. Oncol..

[19] Piccirillo S.G.M., Reynolds B.A., Zanetti N., Lamorte G., Binda E., Broggi G., Brem H., Olivi A., Dimeco F., Vescovi A.L. (2006). Bone morphogenetic proteins inhibit the tumorigenic potential of human brain tumour-initiating cells. Nature.

[20] See S.J., Levin V.A., Yung W.K., Hess K.R., Groves M.D. (2004). 13-cis-retinoic acid in the treatment of recurrent glioblastoma multiforme. Neuro Oncol..

[21] Zhou Y.H., Wu X., Tan F., Shi Y.X., Glass T., Liu T.J., Wathen K., Hess K.R., Gumin J., Lang F. (2005). PAX6 suppresses growth of human glioblastoma cells. J. Neurooncol..

[22] Li D.M., Sun H. (1998). PTEN/MMAC1/TEP1 suppresses the tumorigenicity and induces G1 cell cycle arrest in human glioblastoma cells. Proc. Natl. Acad. Sci. USA.

[23] Gomez-Manzano C., Fueyo J., Kyritsis A.P., Steck P.A., Roth J.A., McDonnell T.J., Steck K.D., Levin V.A., Yung W.A. (1996). Adenovirus-mediated transfer of the p53 gene produces rapid and generalized death of human glioma cells via apoptosis. Cancer Res..

[24] Masserdotti G., Gascon S., Gotz M. (2016). Direct neuronal reprogramming: Learning from and for development. Development.

[25] Gascon S., Masserdotti G., Russo G.L., Gotz M. (2017). Direct Neuronal Reprogramming: Achievements, Hurdles, and New Roads to Success. Cell Stem Cell.

[26] Vierbuchen T., Wernig M. (2011). Direct lineage conversions: Unnatural but useful?. Nat. Biotechnol..

[27] Vierbuchen T., Ostermeier A., Pang Z.P., Kokubu Y., Südhof T.C., Wernig M. (2010). Direct conversion of fibroblasts to functional neurons by defined factors. Nature.

[28] Pang Z.P., Yang N., Vierbuchen T., Ostermeier A., Fuentes D.R., Yang T.Q., Citri A., Sebastiano V., Marro S., Südhof T.C. (2011). Induction of human neuronal cells by defined transcription factors. Nature.

[29] Heinrich C., Blum R., Gascón S., Masserdotti G., Tripathi P., Sánchez R., Tiedt S., Schroeder T., Götz M., Berninger B. (2010). Directing astroglia from the cerebral cortex into subtype specific functional neurons. PLoS Biol..

[30] Zhao J., He H., Zhou K., Ren Y., Shi Z., Wu Z., Wang Y., Lu Y., Jiao J. (2012). Neuronal transcription factors induce conversion of human glioma cells to neurons and inhibit tumorigenesis. PLoS ONE.

[31] Su Z., Zang T., Liu M.L., Wang L.L., Niu W., Zhang C.L. (2014). Reprogramming the fate of human glioma cells to impede brain tumor development. Cell Death Dis..

[32] Pattyn A., Simplicio N., van Doorninck J.H., Goridis C., Guillemot F., Brunet J.F. (2004). Ascl1/Mash1 is required for the development of central serotonergic neurons. Nat. Neurosci..

[33] Casarosa S., Fode C., Guillemot F. (1999). Mash1 regulates neurogenesis in the ventral telencephalon. Development.

[34] Horton S., Meredith A., Richardson J.A., Johnson J.E. (1999). Correct coordination of neuronal differentiation events in ventral forebrain requires the bHLH factor MASH1. Mol. Cell Neurosci..

[35] Chanda S., Ang C.E., Davila J., Pak C., Mall M., Lee Q.Y., Ahlenius H., Jung S.W., Südhof T.C., Wernig M. (2014). Generation of induced neuronal cells by the single reprogramming factor ASCL1. Stem Cell Rep..

[36] Grzmil M., Morin P., Lino M.M., Merlo A., Frank S., Wang Y., Moncayo G., Hemmings B.A. (2011). MAP kinase-interacting kinase 1 regulates SMAD2-dependent TGF-beta signaling pathway in human glioblastoma. Cancer Res..

[37] Edgar R., Barrett T. (2006). NCBI GEO standards and services for microarray data. Nat. Biotechnol..

[38] Tomczak K., Czerwinska P., Wiznerowicz M. (2015). The Cancer Genome Atlas (TCGA): An immeasurable source of knowledge. Contemp. Oncol..

[39] Sun X., Hu X., Wang D., Yuan Y., Qin S., Tan Z., Gu Y., Huang X., He C., Su Z. (2017). Establishment and characterization of primary astrocyte culture from adult mouse brain. Brain Res. Bull..

[40] Gleeson J.G., Lin P.T., Flanagan L.A., Walsh C.A. (1999). Doublecortin is a microtubule-associated protein and is expressed widely by migrating neurons. Neuron.

[41] Brown J.P., Couillard-Després S., Cooper-Kuhn C.M., Winkler J., Aigner L., Kuhn H.G. (2003). Transient expression of doublecortin during adult neurogenesis. J. Comp. Neurol..

[42] Schuurmans C., Guillemot F. (2002). Molecular mechanisms underlying cell fate specification in the developing telencephalon. Curr. Opin. Neurobiol..

[43] Chouchane M., Costa M.R. (2019). Instructing neuronal identity during CNS development and astroglial-lineage reprogramming: Roles of NEUROG2 and ASCL1. Brain Res..

[44] Berninger B., Costa M.R., Koch U., Schroeder T., Sutor B., Grothe B., Götz M. (2007). Functional properties of neurons derived from in vitro reprogrammed postnatal astroglia. J. Neurosci..

[45] Chouchane M., de Farias A.R.M., de Sousa Moura D.M., Hilscher M.M., Schroeder T., Leão R.N., Costa M.R. (2017). Lineage Reprogramming of Astroglial Cells from Different Origins into Distinct Neuronal Subtypes. Stem Cell Rep..

[46] Lee C., Robinson M., Willerth S.M. (2018). Direct Reprogramming of Glioblastoma Cells into Neurons Using Small Molecules. ACS Chem. Neurosci..

[47] Fode C., Ma Q., Casarosa S., Ang S.L., Anderson D.J., Guillemot F. (2000). A role for neural determination genes in specifying the dorsoventral identity of telencephalic neurons. Genes Dev..

[48] Masserdotti G., Gillotin S., Sutor B., Drechsel D., Irmler M., Jørgensen H.F., Sass S., Theis F.J., Beckers J., Berninger B. (2015). Transcriptional Mechanisms of Proneural Factors and REST in Regulating Neuronal Reprogramming of Astrocytes. Cell Stem Cell.

[49] Kim E.J., Battiste J., Nakagawa Y., Johnson J.E. (2008). Ascl1 (Mash1) lineage cells contribute to discrete cell populations in CNS architecture. Mol. Cell Neurosci..

[50] Liu Y., Miao Q., Yuan J., Han S.E., Zhang P., Li S., Rao Z., Zhao W., Ye Q., Geng J. (2015). Ascl1 Converts Dorsal Midbrain Astrocytes into Functional Neurons In Vivo. J. Neurosci..

[51] Kim K., Doi A., Wen B., Ng K., Zhao R., Cahan P., Kim J., Aryee M.J., Ji H., Ehrlich L.I.R. (2010). Epigenetic memory in induced pluripotent stem cells. Nature.

[52] Polo J.M., Liu S., Figueroa M.E., Kulalert W., Eminli S., Tan K.Y., Apostolou E., Stadtfeld M., Li Y., Shioda T. (2010). Cell type of origin influences the molecular and functional properties of mouse induced pluripotent stem cells. Nat. Biotechnol..

[53] Tian C., Wang Y., Sun L., Ma K., Zheng J.C. (2011). Reprogrammed mouse astrocytes retain a “memory” of tissue origin and possess more tendencies for neuronal differentiation than reprogrammed mouse embryonic fibroblasts. Protein Cell.

[54] Ali F.R., Cheng K., Kirwan P., Metcalfe S., Livesey F.J., Barker R.A., Philpott A. (2014). The phosphorylation status of Ascl1 is a key determinant of neuronal differentiation and maturation in vivo and in vitro. Development.

[55] Parras C.M., Schuurmans C., Scardigli R., Kim J., Anderson D.J., Guillemot F. (2002). Divergent functions of the proneural genes Mash1 and Ngn2 in the specification of neuronal subtype identity. Genes Dev..

[56] Imayoshi I., Kageyama R. (2014). Oscillatory control of bHLH factors in neural progenitors. Trends Neurosci..

[57] Jacob J., Kong J., Moore S., Milton C., Sasai N., Gonzalez-Quevedo R., Terriente J., Imayoshi I., Kageyama R., Wilkinson D.G. (2013). Retinoid acid specifies neuronal identity through graded expression of Ascl1. Curr. Biol..

