# Supplementary material for: Inhibition of Glioma Development by ASCL1-Mediated Direct Neuronal Reprogramming
Source: Cells. 2019 Jun 11;8(6):571. doi: 10.3390/cells8060571 (PMC6627512; doi:10.3390/cells8060571)
Supplement: Supplementary file 1 [file cells-08-00571-s001.pdf]

Supplementary Meterial

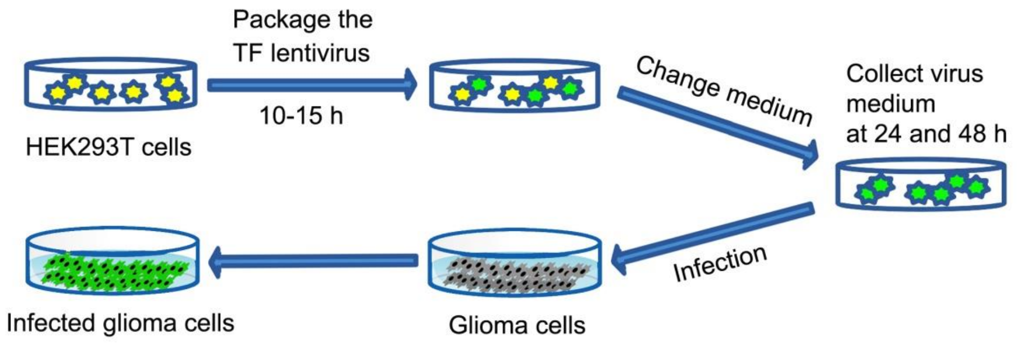

Supplementary Figure 1 Experimental scheme of lentivirus packaging, collection and infection. TF, transcription factor. HEK293T cells (human embryonic kidney cells) were used to package the TF-expressing lentivirus.

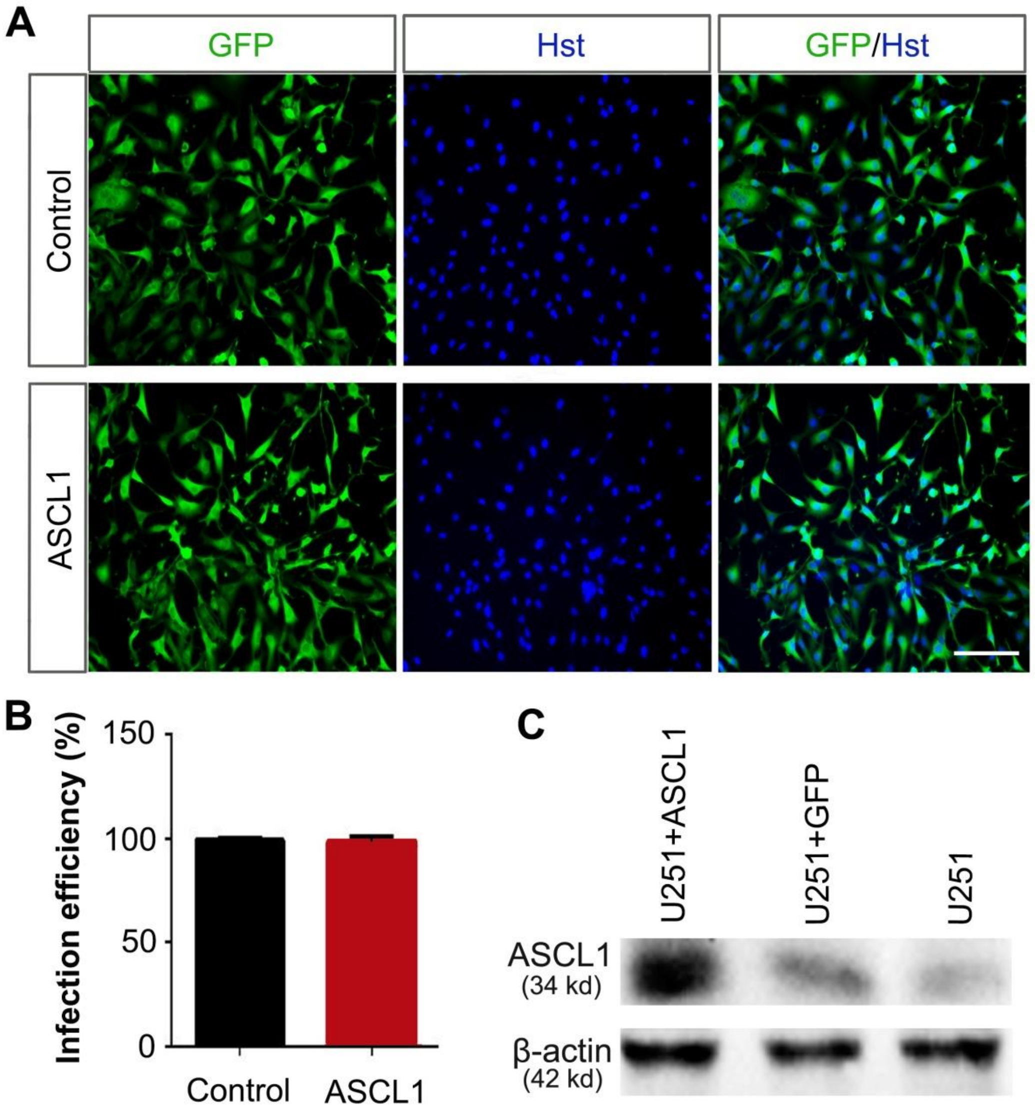

Supplementary Figure 2 Infection of human glioma cells with ASCL1-expressing lentivirus. (A and B) U251 cells were infected with lentivirus expressing ASCL1 or control GFP. At 2 dpi, immunocytochemical analysis was performed to determine the infection efficiency. **(C)** After infection of U251 cells with lentivirus, the ASCL1 expression was confirmed by western blot at 5 dpi. Scales = 100 μm

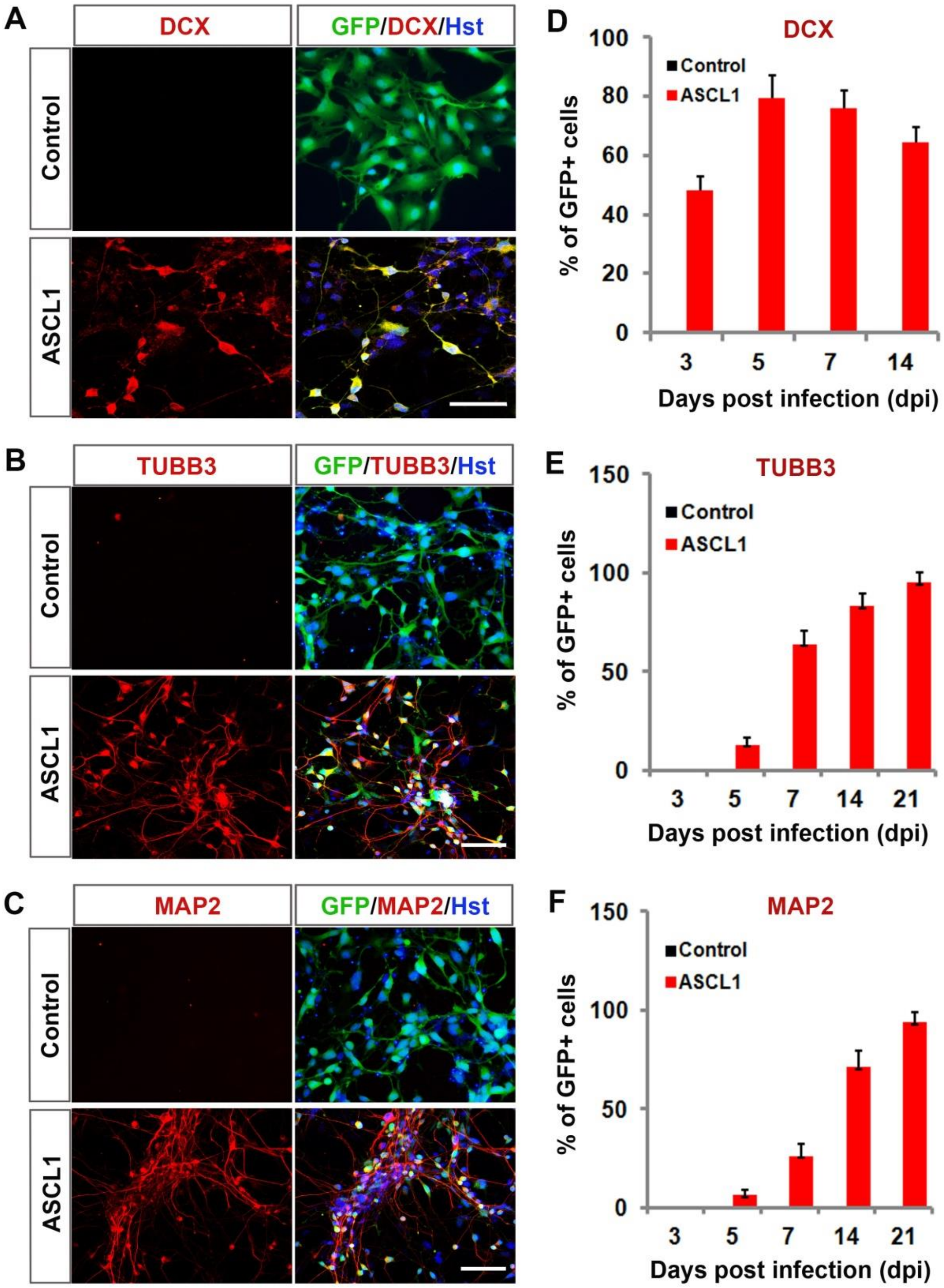

Supplementary Figure 3 Time-course analysis of ASCL1-mediated neuronal reprogramming of U87 human glioma cells. (A-C) Immunostaining of neuronal markers DCX, TUBB3 and MAP2 in ASCL1-converted cells at 14 dpi. **(D-F)** Quantification of neuronal marker expressing in ASCL1-infected U87 cells during the indicated course (n=20 random fields from triplicated samples). Scales: 100 μm.

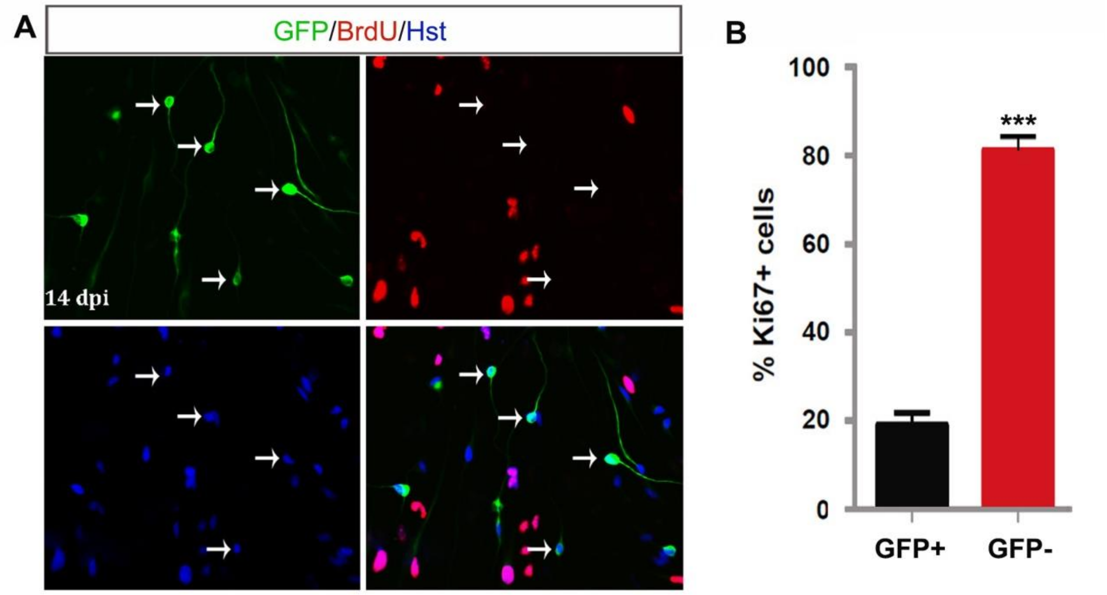

Supplementary Figure 4 Inhibition of glioma cell proliferation by ASCL1-mediated neuronal reprogramming. (A) Immunocytochemical analysis of the proliferation of U251 cells by Ki67 staining at 14 dpi (n=20 randomly selected fields from triplicate samples). Arrows indicate the ASCL1-expressing cells (GFP+) that acquired a neuron-like morphology and were negative for Ki67. **(B)** Quantification of Ki67-positive cells in ASCL1-infected (GFP+) and ASCL1-noninfected (GFP-) group. ****P*<0.001 by Student's *t*-test. Scales = 100 μm.

Supplementary Table 1. Primary antibodies used for immunostaining

Antibody	Host	Dilution	Source	Catalog number
BrdU (5-bromodeoxyuridine)	Rat	1:100	ThermoFisher	MA1-82088
ChAT (Choline acetyltransferase)	Goat	1:100	Millipore	AB144P
DCX (Doublecortin)	Rabbit	1:100	CST	4604S
GABA (γ-aminobutyric acid)	Rabbit	1:100	Sigma	A2052
GFP (Green fluorescent protein)	Chicken	1:600	AVES LABS	1020
Ki67	Mouse	1:100	Abcam	ab6526
	Rabbit	1:100	CST	9129S
MAP2 (Microtubule-associated protein-2)	Rabbit	1:100	Abcam	Ab32454
TUBB3 (Tubulin beta-3)	Mouse	1:100	Biolegend	801202
	Rabbit	1:100	Abcam	AB18207
vGluT1 (Vesicular glutamate transporter 1)	Rabbit	1:100	SYSY	135302
SYN-1 (Synapsin-1)	Rabbit	1:100	CST	5297S
